# Epidemiological and Genomic Analysis of a Large SARS-CoV-2 Outbreak in a Long-Term Care Facility in Catalonia, Spain

**DOI:** 10.1128/msphere.00346-22

**Published:** 2022-11-30

**Authors:** Antoni E. Bordoy, Xavier Vallès, Anna Not, Álvaro Chiner-Oms, Verónica Saludes, Judit Torres Cervós, Anna Roset Roig, Concha Juan-Andres, Helena Sureda, Vanessa Pardo-Amil, Irene García, Gemma Guitart Rossell, Laura Cambra Cibeira, Cristina Casañ, Montserrat Giménez, Ignacio Blanco, Manuela Torres-Puente, Irving Cancino-Muñoz, Fernando González-Candelas, Iñaki Comas, Elisa Martró

**Affiliations:** a Microbiology Department, Laboratori Clínic Metropolitana Nord, Hospital Universitari Germans Trias i Pujol (HUGTiP), Badalona, Barcelona, Spain; b Institut de Recerca Germans Trias i Pujol (IGTP), Badalona, Barcelona, Spain; c SeqCOVID-SPAIN consortium, Valencia, Spain; d Programa de Salut Internacional, Institut Català de la Salut, Barcelona, Spain; e Instituto de Biomedicina de Valencia (IBV-CSIC), Valencia, Spain; f CIBER in Epidemiology and Public Health (CIBERESP), Madrid, Spain; g Fundació Pere Mata, Barcelona, Spain; h Equip d’Atenció Primària Alt Mogent, Direcció d’Atenció Primària Regió Metropolitana Nord, Institut Català de la Salut, Barcelona, Spain; i Àrea de Suport Assistencial, Direcció d’Atenció Primària-Vallès Oriental, Institut Català de la Salut, Barcelona, Spain; j Joint Research Unit “Infection and Public Health” FISABIO-University of Valencia I^2^SysBio, Valencia, Spain; University of Maryland School of Medicine

**Keywords:** SARS-CoV-2, COVID-19, residential facilities, occupational risk, transmission

## Abstract

Limiting outbreaks in long-term care facilities (LTCFs) is a cornerstone strategy to avoid an excess of COVID-19-related morbidity and mortality and to reduce its burden on the health system. We studied a large outbreak that occurred at an LTCF, combining methods of classical and genomic epidemiology analysis. The outbreak lasted for 31 days among residents, with an attack rate of 98% and 57% among residents and staff, respectively. The case fatality rate among residents was 16% (*n *= 15). Phylogenetic analysis of 59 SARS-CoV-2 isolates revealed the presence of two closely related viral variants in all cases (B.1.177 lineage), revealing a far more complex outbreak than initially thought and suggesting an initial spread driven by staff members. In turn, our results suggest that resident relocations to mitigate viral spread might have increased the risk of infection for staff members, creating secondary chains of transmission that were responsible for prolonging the outbreak. Our results highlight the importance of considering unnoticed chains of transmission early during an outbreak and making an adequate use and interpretation of diagnostic tests. Outbreak containment measures should be carefully tailored to each LTCF.

**IMPORTANCE** The impact of COVID-19 on long-term care facilities (LTCFs) has been disproportionately large due to the high frailty of the residents. Here, we report epidemiological and genomic findings of a large outbreak that occurred at an LTCF, which ultimately affected almost all residents and nearly half of staff members. We found that the outbreak was initially driven by staff members; however, later resident relocation to limit the outbreak resulted in transmission from residents to staff members, evidencing the complexity and different phases of the outbreak. The phylogenetic analysis of SARS-CoV-2 isolates indicated that two closely related variants were responsible for the large outbreak. Our study highlights the importance of combining methods of classical and genomic epidemiology to take appropriate outbreak containment measures in LTCFs.

## INTRODUCTION

Most COVID-19 deaths have occurred in people over 75 years old (1) because of various factors, including high frailty due to the existence of many chronic conditions (reviewed in Andrew et al. [2]). In the case of long-term-care facilities (LTCFs), structural and socioeconomic characteristics, being located in urban areas, and a larger capacity have also been reported to affect resident mortality (3, 4). Since March 2020, six epidemic waves have been reported in Spain (5). Nearly half of the total reported deaths during the first wave of COVID-19 (spring of 2020) were from LTCF residents (6). More specifically, in the Northern Metropolitan Area of Barcelona, this metric was 40%, and the total deaths registered during the first wave were close to 20% of the case fatality rate (CFR) among residential population (3, 4) and 18.1% in Catalonia on 27 December 2020 (7). In Spain, vaccination of nursing home residents took place between December 2020 and March 2021. In April 2021, vaccination coverage for this population reached 89% (8). However, the circulation of newly evolved, more transmissible variants, combined with the waning vaccine effectiveness over time against SARS-CoV-2 infection, poses a risk for new outbreaks (9). Thus, understanding and preventing any favorable conditions for the transmission of respiratory-type infectious agents in LTCFs remains important. Furthermore, keeping the residential population safe from SARS-CoV-2 infection indirectly preserves the general well-being (avoidance of lockdowns and isolations) and reduces its burden on the health system. The precise dynamics of the introduction, expansion, and severity of the infection in residential premises are still not well understood, and reports of outbreaks that occurred at these facilities are very scarce, with half of them providing no or very limited SARS-CoV-2 genomic information (10 to 15). Therefore, an in-depth analysis of a specific outbreak using classical and genomic epidemiology approaches may help to answer these questions and guide the implementation of more efficient measures to prevent and mitigate SARS-CoV-2 outbreaks in LTCFs. In that regard, here we studied a large outbreak that was detected on 3 December 2020 at a LTCF in Catalonia, Spain. The outbreak described here took place during the third wave, before vaccination against SARS-CoV-2 infection started. Despite the outstanding success of the vaccination in reducing COVID-19 severity in this highly vulnerable population (7), studies of outbreaks occurring in LTCFs are necessary since the risk for widespread transmission still remains high due to the declining effect of vaccine-induced immunity over time and the emergence of new variants of concern (12, 16).

## RESULTS

### Outbreak development.

**FIG 1:**
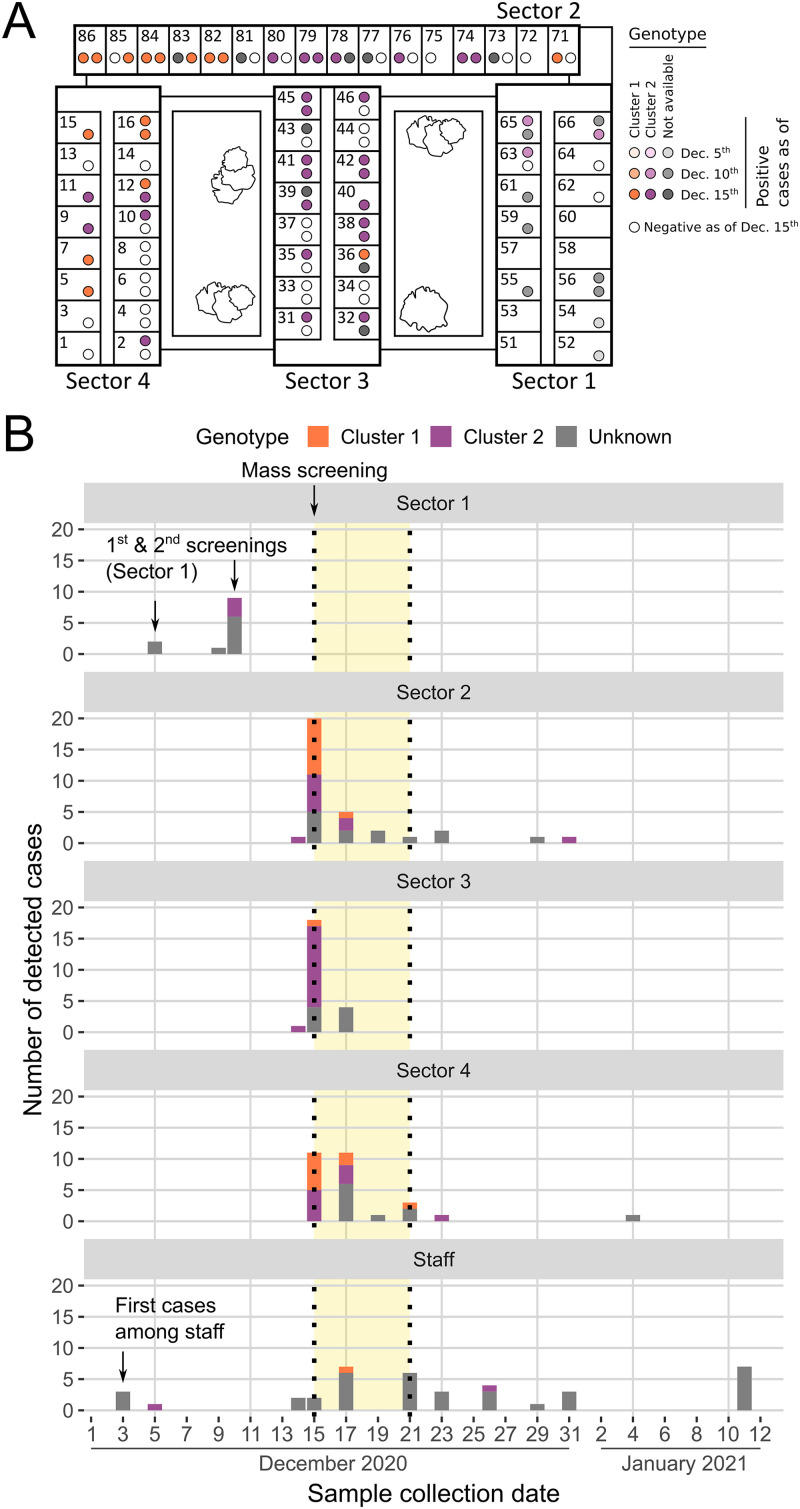
Outbreak development. (A) Ground floor layout of the long-term-care facility. Each sector and room numbers are indicated. The depicted situation corresponds to 15 December 2020. Colors indicate infection status at different time points during the initial phases of the outbreak and the corresponding SARS-CoV-2 cluster. (B) Chronology of outbreak events and cases. For each case detected among residents and workers, the date of sample collection and the SARS-CoV-2 cluster detected are indicated. The period with a yellow background corresponds to the time of resident relocations according to their infection status (phase III of the outbreak).

Fig. 1 summarizes the key events related to the outbreak. On 3 December 2020, routine screening detected SARS-CoV-2 infection in three staff members. Two of them were considered to entail a low risk of transmission for residents due to a previous positive IgG result and a lack of contact with residents, respectively. However, the third staff member had had direct contact with 14 residents located in rooms 51 to 66 of Sector 1 (Fig. 1A). On 5 December, the exposed residents of Sector 1 were screened, detecting two positive cases without roommates in neighboring rooms 52 and 54; staff members were not included in the screening. Additionally, a symptomatic staff member also tested positive on 5 December. Consequently, for the first time since the beginning of the COVID-19 pandemic, an outbreak was declared at this LTCF. On 9 December, a symptomatic resident in room 65 tested positive on a rapid antigen test. On 10 December, after three more residents of Sector 1 developed mild symptomatology, a second screening was conducted on Sector 1, yielding nine new cases (Fig. 1A). Strict measures of biosafety and confinement were then applied to Sector 1. Exposed but not infected residents (*n *= 3) were moved to individual rooms in that same sector.

Following the observation of mild symptoms in several residents, a mass screening of residents and staff was conducted on 14 to 15 December. Four cases were detected on 14 December (two workers and two residents), while 51 new cases were detected on 15 December (2 workers and 49 residents), resulting in an overall attack rate of 64% among residents. Positive residents were spread across all sectors (Fig. 1A and B), with attack rates of 70%, 61%, and 48% for Sectors 2, 3 and 4, respectively. Following existing guidelines, residents were relocated according to their status in new structured and separated sectors and rooms (red: infected; orange: exposed and uninfected; and green: unexposed and uninfected). Sector 4 was the only established green area.

On 17 and 19 December, new screenings were conducted, which yielded 30 additional cases (23 residents, 7 workers). Hence, a total of 86 residents and 15 workers were positive. New systematic screenings were scheduled among the residents who remained negative, with the following results: 4, 3, 1, 1, and 1 new positives detected on 21, 23, 29, and 31 December 2020 and 4 January 2021, respectively, with a final attack rate of 98%. The final CFR was 16% (*n* = 15). After each screening, appropriate resident relocations to the red, orange, or green areas according to their exposure/infection status were carried out until 21 December; thenceforth, only occasional transfers of new positives were performed when necessary. Regarding staff, a total of 39 positive workers were detected between 3 December 2020 and 11 January 2021 (57% of the total active workforce screened, *n *= 68).

### Population characteristics and survival analysis of residents and workers.

**FIG 2:**
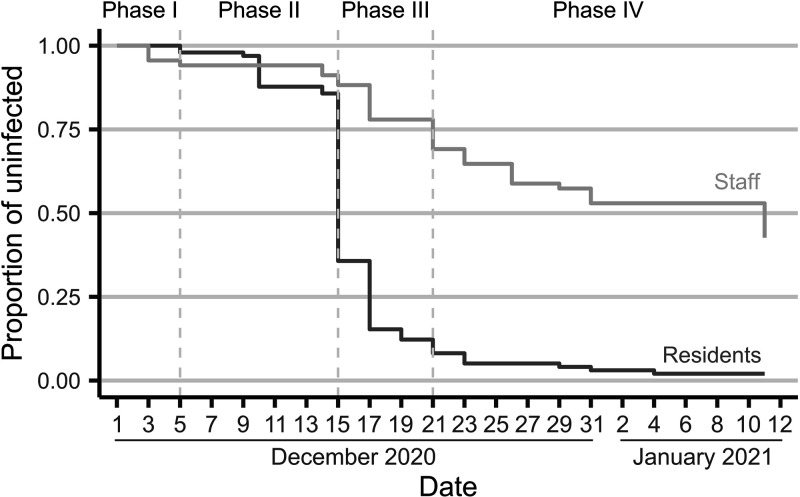
Kaplan-Meier survival estimates. Each phase of the outbreak is indicated. Survival estimate curves for staff (light gray) and residents (dark gray) had parallel trajectories, with a super spreading event (phase II) resulting in a great number of cases among residents, which in turn infected staff members during the relocation period (phase III).

The median age of the residents was 88 years (range, 62 to 107), with 72.4% (*n *= 71) being female, with no differences regarding age distribution. Among facility personnel, median age was 41 (range, 19 to 61). A survival analysis was conducted for both subcohorts (residents and workers); the latter analysis was complemented with an epidemiologic survey among 67 staff members (98.5% active workforce). Fig. 2 shows the survival curves of each subcohort and the phases of the outbreak. Whereas infection among residents was widespread, the attack rate among workers was lower but spanned longer in time. According to the intensity of the transmission and the implemented measures, the outbreak longitudinal evolution could be divided into four different phases: (i) an initial or induction phase (phase I, days prior to 5 December), during which the virus was presumably introduced into the LTCF; (ii) a super spreading phase (phase II, 6 to 14 December); (iii) a relocation phase (phase III, 15 to 21 December); and (iv) a late infection phase (phase IV, days after 21 December).

### Risk factors for infection among staff.

The epidemiological analysis of the staff subcohort (see Table 1) revealed that physical contact with residents was the only significant factor increasing the risk of infection (*P* value < 0.05). More specifically, a quasi-significant association was observed between the staff in charge of patient relocations (*n *= 11) and the risk of infection (*P* value = 0.1). Accordingly, a stratified analysis by outbreak phases revealed that 8 out of 11 workers carrying out relocations were infected during phase III (*P* value < 0.05). Besides, 10 out of 11 workers infected during phase II shared the same dressing room (*P* value < 0.05).

**TABLE 1:** Analysis of epidemiologic survey among staff members

	Total		Infected		Not infected		
Variable	*n*	%	*N*	%	*n*	%	*P* value
Mean age (SD)	40.7 (13.2)		40.8 (11.9)		40.6 (14.6)		0.9
Work position							
Geriatric asst./Geroculture	42	63.6	22	52.4	20	47.6	0.4
Nurse	2	3.0	1	50.0	1	50.0	-
Administrative	3	4.6	1	33.3	2	66.7	-
Laundry	1	1.5	1	100	0	0.0	-
Janitor	6	9.1	0	0.0	6	100.0	-
Technician	3	4.6	2	66.7	1	33.3	-
Other	9	13.6	4	44.4	5	55.6	-
Area							
Yellow	30	45.5	12	40.0	18	60.0	0.2
Green	28	42.2	12	42.9	16	57.1	0.4
Blue	17	25.8	10	58.8	7	41.2	0.3
Red	30	45.5	16	53.3	14	46.7	0.5
Working shift							
Morning shift	36	54.6	18	50.0	18	50.0	0.8
Afternoon shift	21	31.8	10	47.6	11	52.4	0.9
Night shift	5	7.6	2	40.0	3	60.0	0.7
Common areas							
COVID dressing room	45	68.2	23	51.1	22	48.9	0.8
Non-COVID dressing room	19	28.9	9	47.4	10	52.6	
COVID dining hall	33	50.0	17	51.5	16	48.5	0.9
Non-COVID dining hall	15	22.7	8	53.3	7	46.7	
Shared dressing room	35	53.9	18	51.4	17	48.6	0.9
Shared dining hall	35	53.9	19	54.3	16	45.7	0.5
Lived with a colleague	6	9.1	1	16.7	5	83.3	0.2
Shared transportation	2	3.0	0	0.0	2	100.0	0.5
Contact with residents	52	78.9	29	55.8	23	44.2	<0.05
Carried out resident relocations	11	16.7	8	72.3	3	27.8	0.1

### Traceability study of late cases among residents.

The traceability consisted on the reconstruction of room and roommate histories of residents with late infection (phases III and IV). Four case studies were analyzed and are briefly described below. In case 1, the positive test result obtained after 24 days of resident isolation probably reflected the period of clearance of an unnoticed infection. In case 2, repeatedly technically invalid PCR results from one resident who later tested positive probably increased the chances to infect their roommate. Cases 3 and 4 involved eight residents involved in numerous relocations. The traceability study showed that four of the eight residents could have been exposed multiple times to the virus, increasing their overall risk of becoming infected. In any of these four case studies, an unnoticed transmission event from an undetected positive staff member could not be ruled out.

### Whole-genome sequencing results.

**FIG 3:**
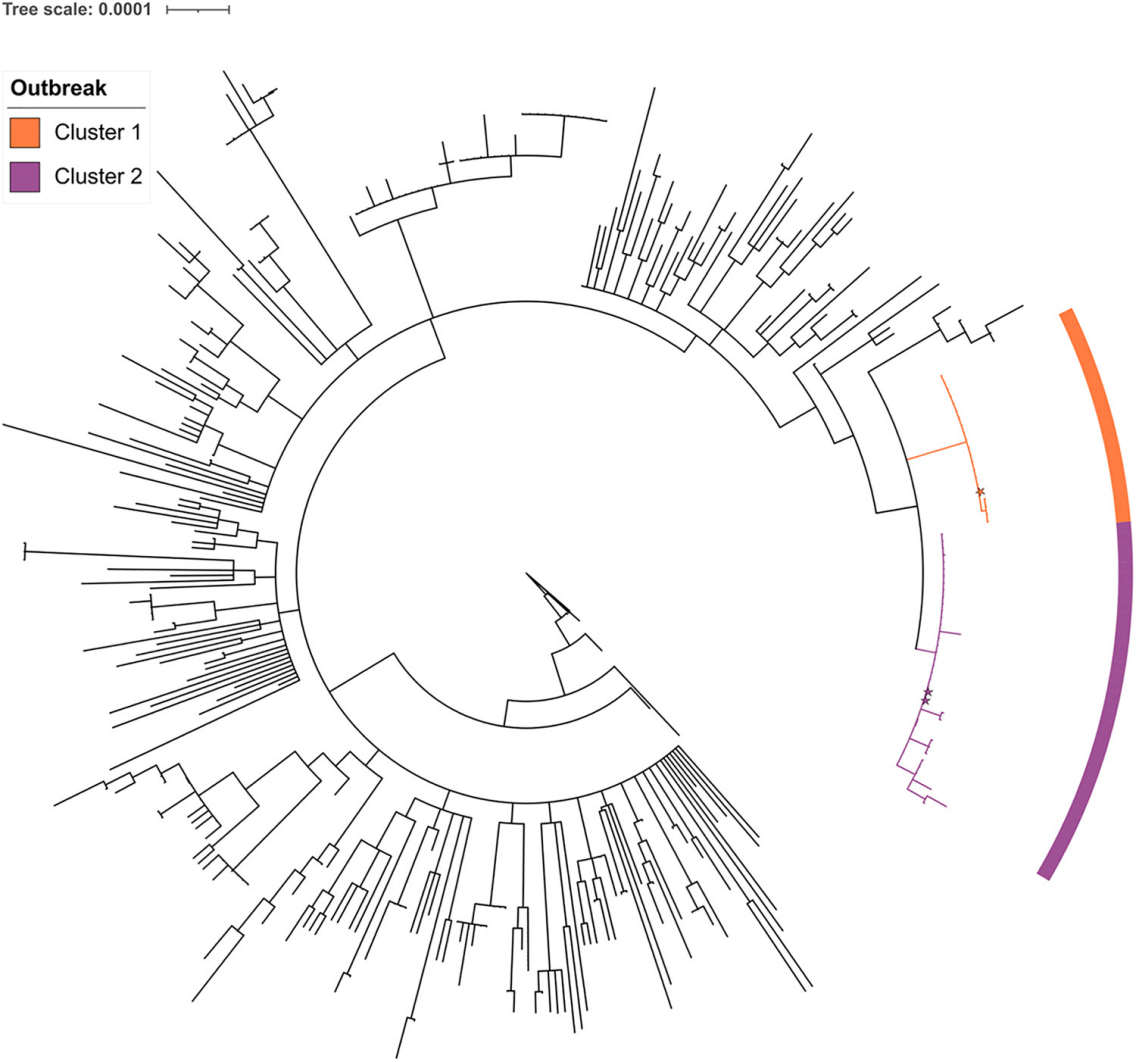
Maximum-likelihood phylogenetic tree. Detail of the rooted phylogenetic tree, including the sequences from our study along with other sequences from Catalonia for the same time period. Shades in the tree branches and corresponding labels indicate each genomic cluster. Star shapes indicate staff members. The scale bar represents the number of substitutions per nucleotide position.

Among available samples from SARS-CoV-2-positive cases (94 samples from 83 unique individuals: 80 residents and 3 staff members), 21 resident samples with *C_T_* (cycle threshold) of >30 and 12 serial resident samples were excluded from whole-genome sequencing (WGS). From the remaining 61 samples, a high sequence quality was obtained for 59 of them, which were included in the phylogenetic study (Fig. 3), accounting for 71.0% (59/83) of available individual samples, which in turn represented 58.3% (56/96) of infected residents and 7.7% (3/39) of infected staff members.

Two different SARS-CoV-2 variants of lineage B.1.177, which was predominant in the general population in Catalonia during that period, were detected, indicating that at least two different viral introductions occurred. Sequences grouped into two closely related clusters with 21 (Cluster 1) and 38 (Cluster 2) sequences, respectively, which differed from each other by at least 3 single nucleotide polymorphisms (SNPs; pairwise average SNPs, 4.3; range, 3 to 7), whereas the average pairwise number of SNPs within each cluster was 0.0 (range 0 to 1) and 0.8 (range 0 to 4), respectively, for Clusters 1 and 2. According to the average SARS-CoV-2 mutation rate (~1 × 10^−3^ substitutions/site/year), ≤2 SNPs are expected for recent transmission events (17). Mutations A9338C (ORF1a:N3025H), C22594T (synonymous), and C29370T (N:T366I) were present in most Cluster 1 sequences, while mutation C15960T (synonymous) was exclusive of Cluster 2 sequences. Cluster 1 was detected for the first time on samples collected during the second screening conducted in Sector 1 during 9 and 10 December. As of 15 December, both B.1.177 clusters were detected in Sectors 2 and 4 in similar proportions, while Cluster 2 was predominant in Sector 3 (Fig. 1). Among workers, two Cluster 2 and one Cluster 1 sequences were observed on 5, 26, and 17 December, respectively. Overall, obtained results point to the existence of two simultaneous transmission networks since the beginning of the outbreak.

## DISCUSSION

This study combines classical and genomic epidemiological analyses to unravel a large outbreak in an LTCF. We reconstructed the most plausible scenario that may explain the large extent and complex dynamics of this outbreak.

The outbreak was most probably initiated by one or more staff members, since (i) visits by relatives were limited and conditioned to strict control measures of social distancing, and (ii) residents of different sectors did not have contact among them after outbreak detection. However, we cannot rule out any of the following possibilities: (i) that one staff member infected by a resident produced the initial spread among staff members, or (ii) that other factors that further fostered transmission, such as the identification of a group of wandering residents located in rooms 84 to 86, could have played a role in the initial spreading within Sector 4 and to staff members. The presence of both B.1.177 clusters in all four sectors indicates the existence of undetected chains of transmission inside the institution before the outbreak was detected or during its evolution, evidencing that the initial focus on Sector 1 was insufficient to control the outbreak, as the real spread of the virus was underestimated. Therefore, genomic epidemiology studies can be especially useful if carried out in near real time when large outbreaks occur.

A very intense relocation of residents took place between 14 and 21 December that significantly increased the risk of infection for staff members (*P* value < 0.05). Therefore, the failure of considering relocations as risky events for transmission combined with the ill-suited management of cases with a negative test result may have contributed to the large extent of the outbreak. However, it is also important to note that social distancing between residents and staff is frequently impossible considering the needs of the residential population. While nonpharmaceutical interventions were reinforced on both residents and staff members, the consistent use of masks among residents was often suboptimal due to a lack of proper understanding of such protective actions by the residents owing to their advanced age and any cognitive disorders. Overall, this makes any close-contact maneuver with a resident a highly risky procedure for bidirectional staff–resident transmission.

Altogether, this study evidences that outbreak containment in LTCFs has inherently associated difficulties. On the one hand, SARS-CoV-2 transmission might be affected by many factors, including mass gatherings, number of social contacts, overcrowding, natural history of the infection, nature of the contacts, circulating viral variant, etc. Importantly, it has also been proposed to be mostly driven by a small percentage of positive individuals (18). Consequently, timely transmission detection will most often be impossible and lead to a situation where guidelines to stop it are not sufficient when such a phenomenon arises, as was the case in this study. Therefore, hasty actions amid an active transmission outbreak may be counterproductive. On the other hand, severity of COVID-19 has been related to the levels of viral load exposure during the transmission event (19), which may be drastically lowered by social distancing and nonpharmaceutical interventions. Regardless of the fact that almost all residents were infected during the outbreak, it is important to underscore that the observed CFR (16%) was far below the one observed during the first wave (up to 40%) (3, 4), with 15 deceased individuals, all of whom had high frailty scores. This rate is similar to the CFR observed among the population over 80 years old infected in the general population (20). Thus, containment measures are important to control not only the spread of the infection but also the severity of the disease, as had been observed during the first wave of the pandemic, when the CFR of infected residents dropped consistently once the biosecurity measures were implemented (4).

For an outbreak that occurred at a Belgian nursing home during December 2020, a cultural event was identified as the starting point of the viral spread of a single variant, and it was found that risk of infection among residents was not significantly associated with close contact or mask wearing (14). The findings of the outbreak reported here, together with those of the Belgian outbreak, show how outbreak dynamics in LTCFs may vary depending on the main routes of transmission of the virus. Another study of a Canadian outbreak in a LTCF during March 2020 reported no cases among new staff brought into the nursing home to cope with a personnel shortage. However, in our study, many workers were infected during phase III (resident relocation). Thus, further studies are needed to determine the professional risk factors of LTCF workers associated with SARS-CoV-2 infection during outbreaks. Even though the outbreak scenario presented here, which took place during the prevaccination period, differs from the current situation, it is important to remark that outbreaks in LTCFs still occur after vaccination deployment (12, 13).

This investigation has a number of limitations. First, we could not determine the directionality of the infections, and we should rely on the most parsimonious assumptions. Second, diagnostic results obtained at least until mid-December 2020 should be considered retrospective data (unknown date of exposition), whereas from this date onwards we were able to actively carry out systematic tests and describe prospectively occurring infections. Therefore, survival curves (Kaplan-Meier) reflect confirmed cases at a given time but not incident cases, especially in phases I and II, due to the fact that most positives were identified through mass screening; thus, they are rather an extrapolation to the true survival curves. Finally, only about half of the samples collected from residents and three samples collected among workers could be sequenced, limiting the ability to test the hypothesis that the outbreak arose among staff members. Moreover, given the rather low viral mutation rate, the possibility that observed cases with highly related genomes represented separate introduction events into the LTCF cannot be completely ruled out (21).

In conclusion, our results suggest that the outbreak was initiated by staff members. Premature mobilization of exposed residents to seemingly unaffected areas led to secondary chains of transmission that prolonged the outbreak. In turn, close physical contact with residents during relocations increased the risk of infection of staff members. However, the infection control guidelines implemented could have contributed to keep mortality at low levels. Our analysis highlights the importance of carefully tailoring measures for outbreak containment to each LTCF.

## MATERIALS AND METHODS

### Place and study population.

The LTCF where the outbreak took place can host 100 residents. At the time of outbreak detection (3 December 2020), occupancy was 98%. The staff team included 70 workers providing different services. At that time, neither residents nor staff members had been vaccinated. Also, visits by relatives were not allowed during this period. In this two-story building, all rooms are located on the ground floor and are distributed in four stand-alone sectors (Fig. 1A) that worked independently, for both residents and staff, to reduce risk of transmission. After outbreak declaration, isolation between sectors included staff members working in contact with residents but excluded the common premises. The basement has several facilities for services and staff that were also considered in the study.

### Study design.

We carried out a prospective cohort study of residential population and exposed staff, including SARS-CoV-2 genomic sequencing of positive samples and the subsequent genomic epidemiology analysis. After outbreak declaration on 4 December 2020, all exposed residents and staff members were screened by molecular tests every 4 days. However, universal screening of residents was not effectively implemented until 14 December and continued until 15 January. During interscreening intervals, symptomatic residents and workers were tested *ad hoc*. An epidemiological questionnaire, including behavioral and risk factors associated with transmission of SARS-CoV-2, was provided to health staff.

### Sample collection and diagnosis of SARS-CoV-2.

Naso-pharyngeal swabs (NPSs) were collected by trained personnel, and an *in situ* rapid antigen test (Panbio COVID-19 Ag Rapid Test Device, Abbott Diagnostics, Jena, Germany) or laboratory molecular test (reverse transcription PCR, RT-PCR [Allplex 2019-nCoV assay, Seegene, Seoul, South Korea] on CFX96 instruments [Bio-Rad Laboratories] or transcription-mediated amplification, TMA [Grífols S.A., Sant Cugat del Vallès, Spain]) was used to detect the presence of SARS-CoV-2 infection. For molecular diagnosis, samples were shipped to the reference laboratory and stored at 4°C.

### SARS-CoV-2 whole-genome sequencing.

All SARS-CoV-2-positive samples initially processed by TMA were subjected to real-time RT-PCR to determine the cycle threshold (*C_T_*) value. Only those samples with *C_T_* of <30 for the N gene target were selected for whole-genome sequencing (WGS) to maximize sequencing success rate. Genomic retro-transcription, amplification, and sequencing were performed as previously described (22). The ARTIC network v3 amplicon panel (Integrated DNA Technologies) was used for amplification, the Illumina DNA Prep kit for library preparation (Illumina), and the Illumina MiSeq platform for sequencing.

### Bioinformatics and phylogenetics analysis.

Analysis of raw sequencing data was performed with a previously developed pipeline based on IVAR (https://gitlab.com/fisabio-ngs/sars-cov2-mapping) to obtain consensus nucleotide sequences (22). Amino acid substitutions and deletions were identified with Nextclade v0.14.1 (https://clades.nextstrain.org/). The sequences generated were deposited in the GISAID database (https://www.gisaid.org/; accession numbers can be found at https://epicov.org/epi3/epi_set/221123wf). Only samples with genome coverage of >90% were included in the study. A maximum likelihood phylogenetic tree was built along with other sequences circulating in Catalonia between 15 November 2020 and 15 January 2021 obtained from GISAID (*n* = 429) (21, 22). Sequences were aligned against the SARS-CoV-2 reference genome (23) using MAFFT (24). Specific positions that have been reported to be problematic for phylogenetic reconstruction (25) were masked using the mask_alignment.sh script (26). Single nucleotide polymorphisms (SNPs) exclusive of outbreak samples were confirmed by manual Integrative Genomics Viewer inspection (27). Finally, a maximum-likelihood (ML) phylogeny was reconstructed using IQ-TREE 2 (28) with the general time reversible (GTR) model and based on the complete masked genome alignment. This phylogeny was rooted to the SARS-CoV-2 sequence obtained in Wuhan on 24 December 2019 (29). Additionally, the pairwise number of SNPs was calculated between outbreak sequences.

### Statistical analysis.

Epidemiological data were described using means and standard deviation (SD) if quantitative after testing for normal distribution (skewness and kurtosis test) or medians and interquartile ranges (IQR) for continuous variables and proportions for categorical variables. Kaplan-Meier curves were used to estimate the cumulative proportion of exposed participants who were infected, stratified by residents and staff members. Chi-square test was used for bivariate analysis between categorical variables and Student’s *t* test between continuous variables or their nonparametric counterpart, as appropriate (Fisher’s test or Wilcoxon test). A *P* value of ≤0.05 was considered statistically significant. The statistical package Stata 14.0 (StataCorp) was used for analysis.

### Ethics approval.

Informed consent was not required as this study was performed in the context of the SARS-CoV-2 surveillance system in Catalonia.
